# Integrated analysis of lncRNA and mRNA in liver of *Megalobrama amblycephala* post *Aeromonas hydrophila* infection

**DOI:** 10.1186/s12864-021-07969-5

**Published:** 2021-09-11

**Authors:** Qianhui Sun, Jixiu Wang, Guowen Wang, Huanling Wang, Hong Liu

**Affiliations:** 1 College of Fisheries, Key Lab of Freshwater Animal Breeding, Ministry of Agriculture and Rural Affair / Key Lab of Agricultural Animal Genetics, Breeding and Reproduction of Ministry of Education, Huazhong Agricultural University, Wuhan, 430070 China; 2 Engineering Research Center of Green Development for Conventional Aquatic Biological Industry in the Yangtze River Economic Belt, Ministry of Education, Wuhan, 430070 China

**Keywords:** LncRNAs, Immune response, Bacterial infection, *M. amblycephala*, Teleost, ceRNA

## Abstract

**Background:**

As non-coding RNA molecules of more than 200 bp in length, long non-coding RNAs (lncRNAs) play a variety of roles in biological processes, including regulating the immune responses to bacterial infections. In recent years, there have been many in-depth studies on mammalian lncRNAs, but the relevant studies in fish are very limited. Meanwhile, since lncRNAs are not conserved among species, it is difficult to apply the existing results directly to unstudied species.

**Results:**

To obtain the information of lncRNAs in *Megalobrama amblycephala*, one of the most economically important freshwater fish in China, also to better understand the biological significance of lncRNAs in the immunity system, the fish liver at 0, 4, 12, 24, and 72 h post *Aeromonas hydrophila* infection (hpi) were obtained for lncRNA-sequencing (lncRNA-seq). A total of 14,849 lncRNAs were identified, and 2196 lncRNAs showed significant differences at different time points post *A. hydrophila* infection. Gene Ontology (GO) annotation and Kyoto Encyclopedia of Genes and Genomes (KEGG) pathway analyses showed that the target genes of the differentially expressed lncRNAs were enriched in several pathways related to immune such as apoptosis, inflammation, and immune response. Time-specific modules were then identified, using weighted correlation network analysis (WGCNA), and 28 modules significantly correlated with different time point after infection were found. Furthermore, four immune-related genes and six lncRNAs in the time-specific modules were subsequently verified by RT-qPCR.

**Conclusions:**

The above findings reveal the discovery of widespread differentially expressed lncRNAs in the *M. amblycephala* liver post *A. hydrophila* infection, suggesting that lncRNAs might participate in the regulation of host response to bacterial infection, enriching the information of lncRNAs in teleost and providing a resources basis for further studies on the immune function of lncRNAs.

**Supplementary Information:**

The online version contains supplementary material available at 10.1186/s12864-021-07969-5.

## Background

Long noncoding RNAs (lncRNAs) are operationally defined as non-coding RNA molecules with a length of more than 200 base-pairs (bp) [1]. LncRNAs include intergenic lncRNAs (lincRNAs), antisense lncRNAs, intronic lncRNAs, and sense lncRNAs [2]. As multifunctional molecules, lncRNAs play important roles in multiple biological processes by interacting with RNA, DNA, or proteins to change the expression of protein-coding genes [3], which can regulate diverse cellular processes, including disease process, development and cell proliferation [4].

Since the expression pattern of lncRNAs is closely related to the development of many diseases [5, 6], lncRNAs have gradually become a research hotspot in the field of life sciences. LncRNAs regulate gene expression at the epigenetic, transcriptional and post-transcriptional levels [7] and previous studies have shown that they play an important regulatory role in the innate immune system [8, 9]. The adaptive immune cells provide vital immune protection under the guidance of lncRNAs [3]. Meanwhile, microRNAs (miRNAs) can regulate gene expression post-transcriptionally [10]. Bacterial infection in mammals can interferes with miRNA expression, thus altering the mechanism of immune signal transduction, autophagy, or apoptosis [11]. As competing endogenous RNA (ceRNA), messenger RNAs, transcribed pseudogenes, and lncRNAs form a large-scale regulatory network across the transcriptome to communicate with each other through miRNA response elements (MREs) [12]. The MREs in coding and non-coding transcripts affect the expression levels and activities of different ceRNAs [13], which were associated with a variety of diseases [14]. As miRNA sponges, lncRNAs play a role in immunity by competitively inhibit the ability of miRNAs to interact with their mRNA targets [15, 16].

Most studies about immune-related lncRNAs are mainly focused on mammalian species, especially human and mouse. Previous research has established that lncRNAs play a paramount role in immunity by inducing immune gene expression, regulating cytokine genes, adaptive immune cells, and participating in RNA-protein, RNA-DNA, or RNA-RNA interactions [3]. The lncRNA transcripts significantly enrich in autoimmune and immune-related disorders (AID) loci [17], which makes lncRNAs a biomarker for human disease or a target for medical detection. For example, lncRNAs are involved in the host response to viral infection and innate immunity during SARS-CoV infection in mouse [18]. Li et al. (2018) find that lncRNA MEG3–4 is more competitive than miR-138 when combining with proinflammatory cytokine interleukin-1β (*IL-1β*), thus intensifying the inflammatory responses to bacterial infection in mice [19]. Additionally, it’s shown that lncRNAs are specifically involved in the mammalian cell response toward bacterial infections [11].

Due to the low evolutionary conservation of lncRNAs across species [20], the research result of lncRNAs in mammalian species can hardly be applied to aquaculture species. Up to now, a few studies have attempted to explain the role of lncRNA in teleost. When exposed to β-diketone antibiotics (DKAs), the lncRNAs in zebrafish (*Danio rerio*) are abnormally expressed and their potential target genes might play roles in immunity [21]. Besides, antisense lncRNA PU.1 is found to be involved in the adaptive immunity of zebrafish [22]. After infected with *Flavobacterium psychrophilum*, lncRNAs mediate anti-bacterial immune response in rainbow trout (*Oncorhynchus mykiss*) [23]. RNA sequencing of Atlantic salmon (*Salmo salar*) infected with *Piscirickettsia salmonis* suggests that lncRNAs are associated with the genes of endocytosis and iron homeostasis, such as clathrin, hepcidin, and haptoglobin [24].

*Aeromonas hydrophila*, the main pathogen that causes bacterial septicemia in freshwater fish, can lead to high mortality and bring about serious economic losses to the freshwater aquaculture industry. *Megalobrama amblycephala* is an important economic freshwater fish in China, meanwhile it is sensitive to *A. hydrophila* and can be seriously harmed by bacterial septicemia. The liver has the functions of secreting bile, detoxifying, and storing glycogen, and is one of the most core organs for the body to maintain physiological functions. At the same time, the liver has been generally accepted as a major immune organ in teleost [25–27]. As in mammals, hepatocytes are the prime source of acute phase response in fish, and that pro-inflammatory cytokines induce transcription of their genes [28]. Given that lncRNA is an essential part of the immunological process and related knowledge in fish is quite limited, herein, we conducted a comprehensive lncRNAs sequencing in the liver of *M. amblycephala* post *A. hydrophila* infection and performed functional annotation analysis based on the Gene Ontology (GO) and Kyoto Encyclopedia of Genes and Genomes (KEGG) databases. We identified the co-localization of lncRNAs and genes at different time points post infection by using WGCNA (weighted correlation network analysis). This research will enrich the lncRNA database of teleost and contribute to a better understanding of the role of lncRNAs in the immune response of teleost.

## Results

### Identification and characterization of mRNA and lncRNA in the *M. amblycephala* liver

**Fig. 1:**
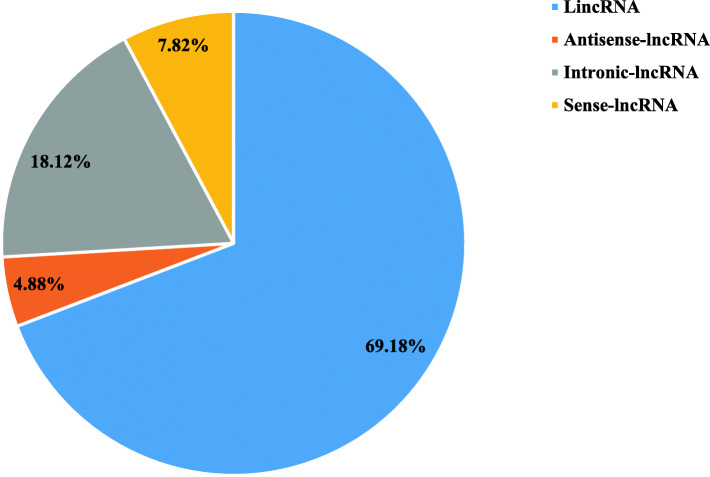
Percentage of different types of predicted lncRNAs in the *Megalobrama amblycephala* liver post *Aeromonas hydrophila* infection

To identify lncRNAs expressed in the *M. amblycephala* liver at different time points after *A. hydrophila* infection, 15 cDNA libraries (5 time points, 3 repetitions per time point) were constructed and sequenced. Clean reads totaling 163.21 Gb were obtained. The mean GC content of the 15 libraries was 48.23% and the Q30 of each sample was no less than 95.68%, suggesting that the sequencing data was highly reliable. On average, 67,576,141 mapped reads were obtained from the clean data (Additional files 1 and 2: Table S1 and S2). A total of 9011 differentially expressed genes (DEGs) were identified. The intersection of the CPC, CNCI, Pfam, and CPAT finally yielded 14,849 lncRNA transcripts, which were classified as 10,272 lincRNAs, 2691 intronic lncRNAs, 1161 sense lncRNAs, and 725 anti-sense lncRNAs (Fig. 1).

To study the basic features of lncRNAs in the *M. amblycephala* liver, lncRNAs were identified and aligned with mRNAs. The results showed that there were 12,689 lncRNAs corresponding to 887,446 target genes (Additional file 3: Table S3). Furthermore, several lncRNAs acting in trans were found to target protein-coding genes specifically related to innate immunity, including interleukin 6 (*IL 6*), hepcidin, transferrin, and complement C3. Some mRNAs (complement C3) are regulated by multiple lncRNAs (e.g. MSTRG.9317.1 and MSTRG.104056.1), and a single lncRNA (MSTRG.97410.1) can target multiple mRNAs (*IL 6*, *NF-kB2*, *TRAF2*, and hepcidin), indicating the functional intersection of these lncRNAs and their common potential target genes.

**Fig. 2:**
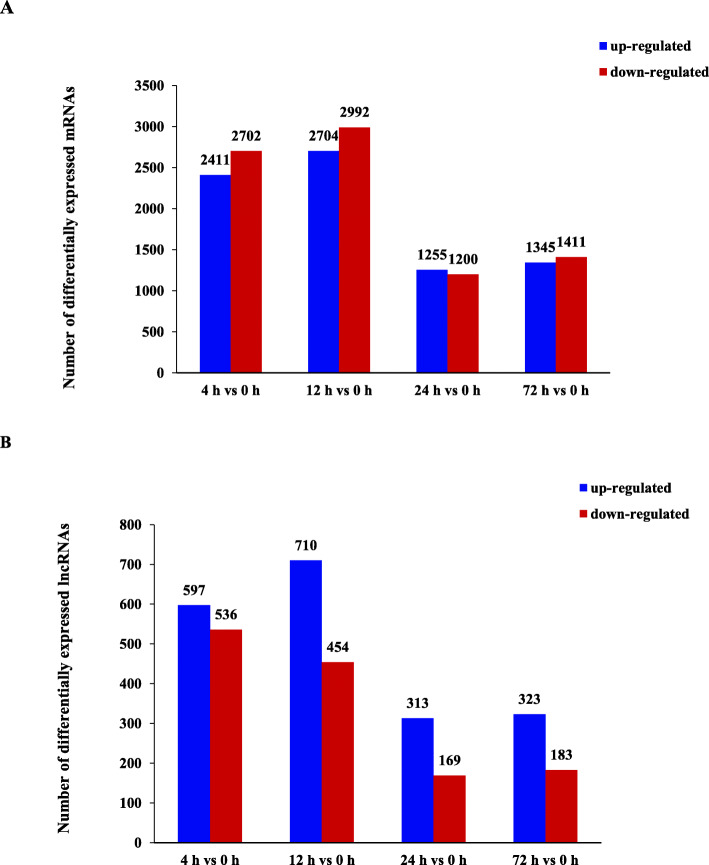
Number of differentially expressed mRNAs (**A**) and lncRNAs (**B**) in the *Megalobrama amblycephala* liver post *Aeromonas hydrophila* infection. The blue and red columns represent significantly up- and down-regulated, respectively, and the number at the top of the column indicates the number of differentially expressed mRNAs or lncRNAs

**Fig. 3:**
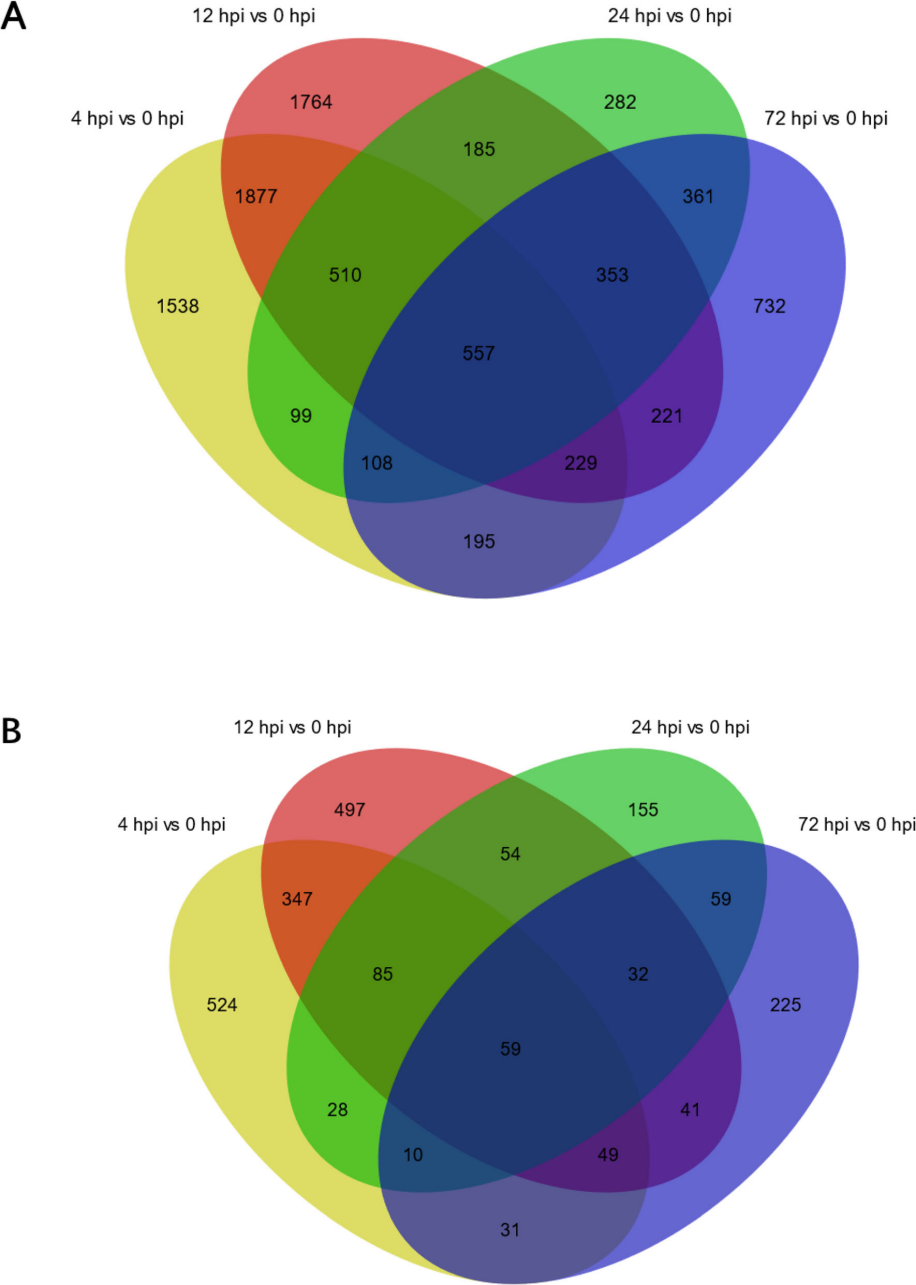
Venn diagram of differentially expressed mRNAs (A) and lncRNAs (B) among four comparisons in the *Megalobrama amblycephala* liver post *Aeromonas hydrophila* infection. Yellow: 4 hpi vs 0 hpi; red: 12 hpi vs 0 hpi; green: 24 hpi vs 0 hpi; and blue: 72 hpi vs 0 hpi. The numbers on the diagram represent the number of differentially expressed mRNAs or lncRNAs that overlap between one or two to four comparisons

**Fig. 4:**
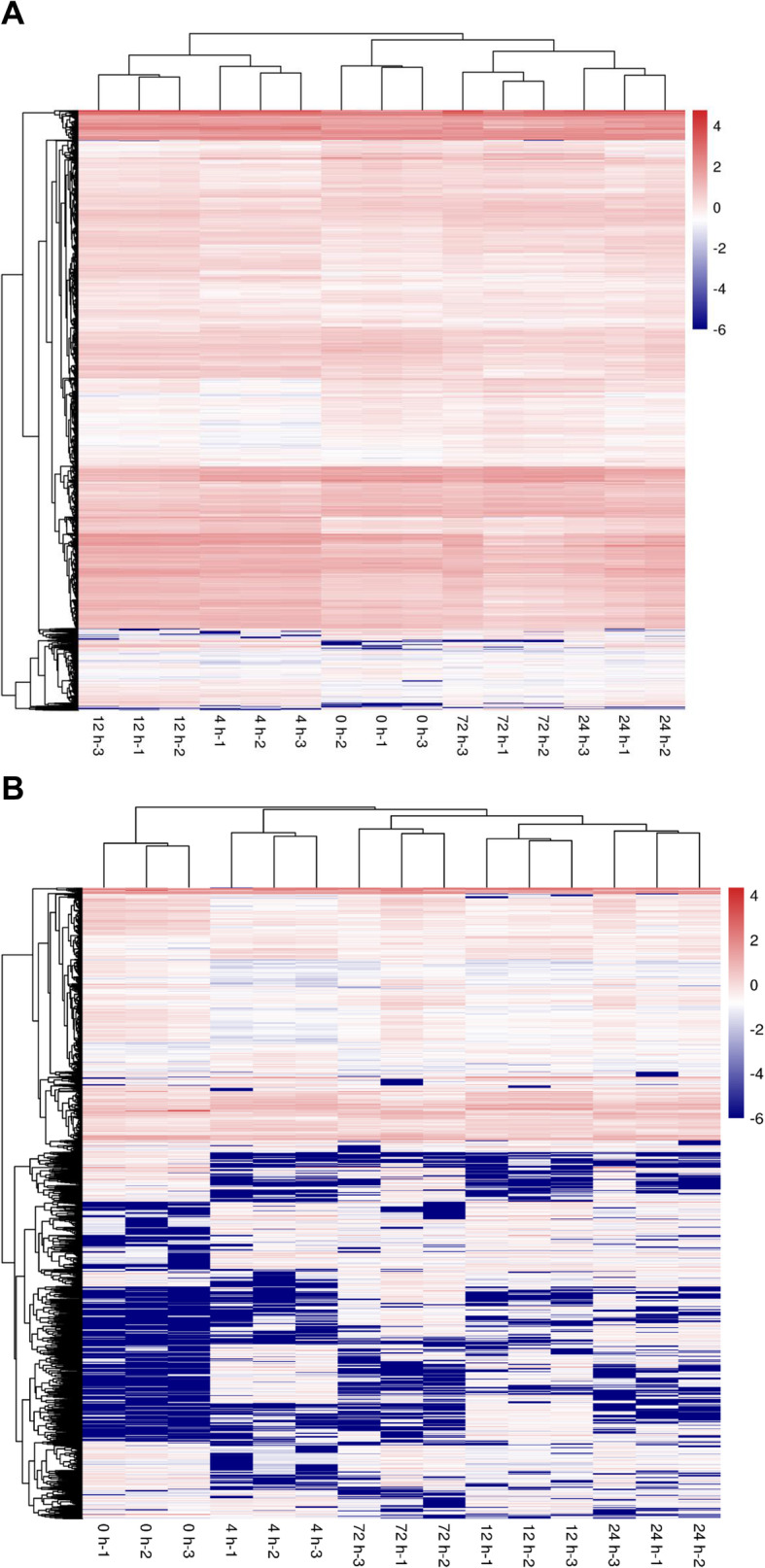
Expression profile of DEGs and DE lncRNAs in the *Megalobrama amblycephala* liver post *Aeromonas hydrophila* infection based on RNA-seq. (**A**) Heatmap of expression profile for the DEGs that showed significant expression changes. (**B**) Heatmap of expression profile for the DE lncRNAs that showed significant expression changes. Red: relatively high expression level; Blue: relatively low expression level. The darker the red color, the higher the expression level, and the darker the blue color, the lower the expression level

Compared to 0 h, there were 1133 differentially expressed lncRNAs (DE lncRNAs) at 4 hpi, 1164 DE lncRNAs at 12 hpi, 482 DE lncRNAs at 24 hpi, and 506 DE lncRNAs at 72 hpi, respectively (FDR < 0.05 and |log2 (Fold Change) | ≥ 1) (Fig. 2). To further analyze the interactions among different time points, we constructed a Venn diagram using the DEGs and DE lncRNAs that were differentially expressed in comparisons of 4 hpi vs 0 hpi, 12 hpi vs 0 hpi, 24 hpi vs 0 hpi, and 72 hpi vs 0 hpi, respectively. A total of 557 overlapping sequences from 9011 DEGs and 59 overlapping sequences from 2196 DE lncRNAs were identified among all the 4 comparisons (Fig. 3). Heat maps indicated overt different clusters before and after *A. hydrophila* infection (Fig. 4). Notably, the results showed a relatively large difference in mRNAs and lncRNAs expression trends among different time points although the time difference was only a few hours.

### GO and KEGG enrichment analyses of target genes of DE lncRNAs

Based on the GO database, the target genes of DE lncRNAs were assigned to the biological processes, cellular components, and molecular function, respectively. The significantly enriched GO terms for DEGs and target genes of DE lncRNAs in each comparison between different time points were shown in Additional files 4 and 5 (Table S4 and S5). In the four comparisons, the significant GO terms of the DEGs were mainly associated with “I-kappaB kinase/NF-kappaB signaling”, “hydrolase activity”, “oxidation-reduction process”, “ncRNA processing”, “nuclear ncRNA surveillance”, “response to metal ion”, “cofactor binding”, “lipid biosynthetic process”, and so on (Additional file 4: Table S4). In contrast, no significant pathway of the target genes of DE lncRNAs was identified at 24 and 72 hpi. The significant GO terms of the target genes were mainly associated with “catalytic activity”, “endoplasmic reticulum cytoplasm”, and “golgi apparatus” at 4 and 12 hpi (Additional file 5: Table S5).

KEGG were performed to understand the enrichment of the lncRNA target genes and to reveal the functions of the DE lncRNAs. KEGG analysis demonstrated that most of the significantly enriched pathways of the target genes of DE lncRNAs post infection belong to the pathways including “adipocytokine signaling pathway”, “herpes simplex infection”, “NOD-like receptor signaling pathway”, “PPAR signaling pathway”, “protein export”, “protein processing in endoplasmic reticulum”, “RIG-I-like receptor signaling pathway”, “TGF-beta signaling pathway”, and “Toll-like receptor signaling pathway” (Additional file 6: Table S6).

### Weighted correlation network analysis (WGCNA) and RT-qPCR validation

**Fig. 5:**
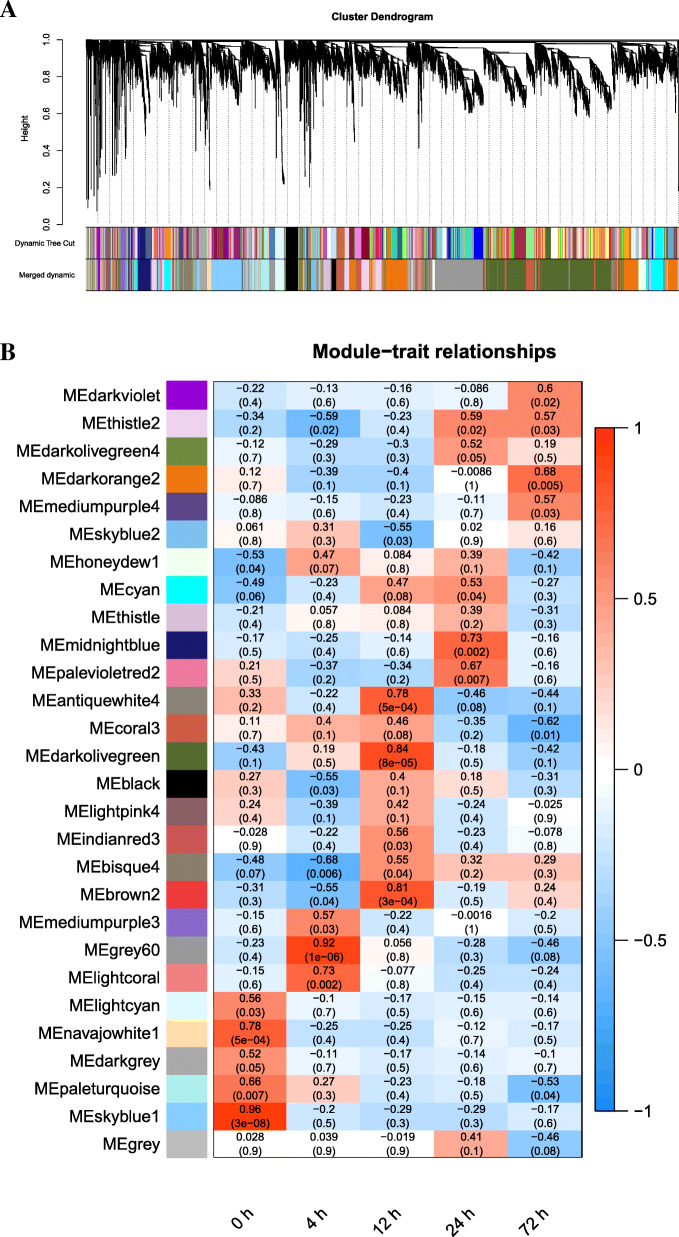
Weighted gene co-expression network analysis (WGCNA) of mRNAs and lncRNAs in the *Megalobrama amblycephala* liver post *Aeromonas hydrophila* infection. (**A**) The dendrogram of all genes is clustered based on a dissimilarity measure (1-TOM). Each single leaf in the tree represents a single gene, the major tree branches constitute 28 distinct modules and are shown in different colors. (**B**) Heatmap of the module-trait relationships. Each row corresponds to a module, and each column represents the specific time points after infection with *Aeromonas hydrophila*. The right color panel represents Pearson’s r correlation coefficient. Red for positive correlation and blue for negative correlation. Each cell contains the corresponding correlation and *p*-value

We combined all the expression matrix of both protein-coding genes and lncRNAs as the input file for WGCNA to identify modules. After excluding deletion and outlier values, an expression matrix of 13,460 transcripts (including 916 lncRNAs and 12,544 protein-coding genes) were obtained for further analysis. We explored and identified the correlations among modules according to clustering analysis. The resulting gene dendrograms and respective module with different colors were shown in Fig. 5A and 28 modules were found to be significantly correlated with different time point post infection (Fig. 5B).

**Fig. 6:**
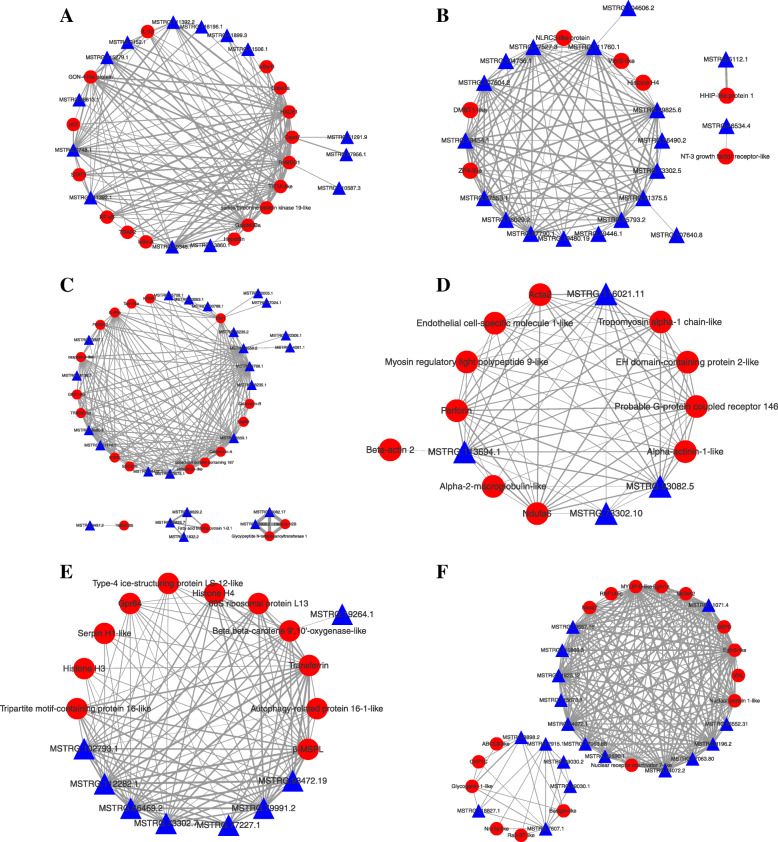
Visualization of connections between mRNAs and lncRNAs in various modules. **A**–**F**: Connections between mRNAs and lncRNAs in grey60 (**A**), darkorange2 (**B**), darkolivegreen (**C**), darkviolet (**D**), mediumpurple3 (**E**), and midnightblue (**F**) modules. The lncRNAs and their corresponding target genes were used to construct the lncRNA–gene interaction network. In this network, red-colored nodes represent mRNAs and blue-colored nodes represent lncRNAs. Define the width of the line according to the weight of node. The higher the weight of the node, the thicker the connecting line, and the stronger the correlation between lncRNAs and mRNAs

Six modules (*P* < 0.05), which were significantly positively correlated with different time points post infection were selected for co-expression analysis (Fig. 6). Grey 60 (*r* = 0.92, *P* = 1e-06) and mediumpurple 3 (*r* = 0.57, *P* = 0.03) modules, which included many immune related genes such as *HACE1*, *IL-1β*, *NF-kB*, transferrin, *p53*, hepcidin and so on were correlated with 4 hpi. The genes such as *Derl1*, *Hsp90*, and calreticulin-like in the darkolivegreen (*r* = 0.84, *P* = 8e-05) module, which might play important roles at 12 hpi, were enriched in the “protein processing in the endoplasmic reticulum” pathway. The midnightblue module (*r* = 0.73, *P* = 0.002) included genes such as egl-9 family hypoxia-inducible factor 1α were correlated with 24 hpi, whereas the darkviolet (*r* = 0.6, *P* = 0.02) and darkorange 2 (*r* = 0.68, *P* = 0.005) modules which included perforin, *NLRC*3-like, and *DMBT*1-like genes, might play important roles at 72 hpi.

**Fig. 7:**
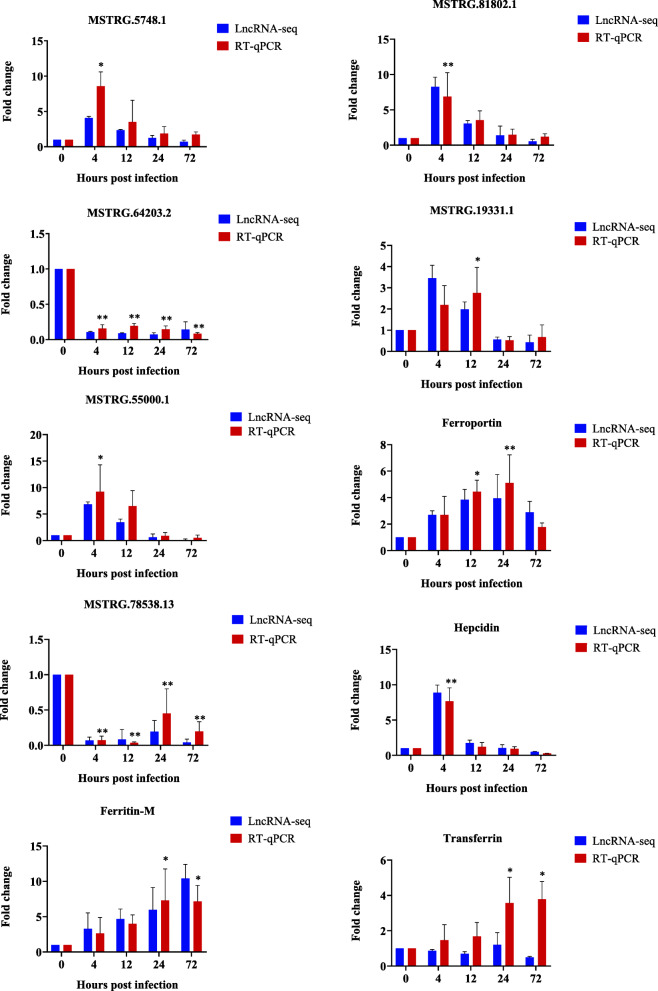
RT-qPCR validation of six differentially expressed lncRNAs and four differentially expressed genes selected. The blue column represents the results of lncRNA-seq, and the red column represents the results of RT-qPCR. Values were described as mean ± SEM (*n* = 3 pools, with 3 fish per pool). Differences were determined by one-way analysis of variance (ANOVA). The asterisks indicate statistically significant differences (*, *P* < 0.05; **, *P* < 0.01)

RT-qPCR was used to verify expression of the selected six DE lncRNAs and four DE genes. The results showed that the expression trends of lncRNAs and target genes identified by high-throughput sequencing were consistent with those identified by RT-qPCR (Fig. 7). The expression of MSTRG.55000.1 and MSTRG.81802.1 was up-regulated at 4 hpi and gradually down-regulated after 12 hpi. The hepcidin gene trans-regulated by those lncRNAs has a similar expression trend. This indicates that lncRNAs have complex functions.

## Discussion

Although there have been many in-depth studies in mammals, the study of fish lncRNA is very inadequate and the results of the former cannot be applied to the latter due to the non-conservativeness of lncRNAs. Several studies indicate that lncRNAs are involved in the immune regulation of teleost fish. For example, of the 5636 non-overlapping lncRNA loci identified, 3325 are differentially expressed during ISA virus (ISAV) infection in the liver of Atlantic salmon [29]. A total of 11,462 lncRNAs are expressed in the liver of Atlantic salmon infected with *Piscirickettsia salmonis*, of which 993 lncRNAs are differentially expressed [30]. There are 8463 lncRNAs identified in the spleen of *Larimichthys crocea* infected with *vibrio parahaemolyticus* [31]. Consistently, in this study, lncRNA-seq was performed on the *M. amblycephala* liver at 0, 4, 12, 24, and 72 hpi post *A. hydrophila* infection. A total of 14,849 lncRNAs were identified, of which 2196 lncRNAs were differentially expressed. The dynamic changes of mRNAs are closely related to the physiological status of fish. Like mRNAs, lncRNAs may have spatial and temporal expression with potentially important roles during bacterial infection. Meanwhile, the expression of lncRNAs in the *M. amblycephala* liver showed significant difference among different time points post challenge. After challenge with *A. hydrophila*, the mortality rate and pathological damage of *M. amblycephala* peaked at 48 hpi and then gradually recovered [32]. We assume that in the early stages post challenge, bacteria proliferated rapidly, and the innate immune of the body quickly responded to kill bacteria. In the late stages post challenge, the immune system continued to kill bacteria in the survived fish, and the bacteria were completely defeated finally. Overall, with dynamic changes of competition between the proliferation of bacteria and the killing bacteria of the immune system, lncRNAs also presented a very dynamic change in the short-term post challenge. The RT-qPCR results further validated that the expression of the DE lncRNAs was consistent with the sequencing data.

There are different kinds of lncRNAs such as lincRNA, intronic lncRNA, sense lncRNA, and antisense lncRNA. It has been reported that most lincRNAs are more likely to act *in cis* through transcriptional interference [2]. Cis-natural antisense lncRNAs may regulate gene expression at the transcription level. The large number of intronic lncRNAs may be pre-mRNA fragments [33], which are transcribed and may encode exons within rarely-expressed transcripts [2]. Among the obtained lncRNAs in *M. amblycephala*, 69.2% were lincRNA, 18.1% were intronic lncRNA, 7.8% were sense lncRNA, and 4.9% were antisense lncRNA. Multiple types of lncRNAs coexisted in this study, and lincRNAs accounted for the largest proportion, indicating that lncRNAs may play multiple biological functions through multiple pathways, and the role of lincRNAs may be the most important.

The DEGs and target genes of DE lncRNAs at different time points were analyzed separately here. GO analysis showed that both DEGs and target genes of DE lncRNAs were mostly enriched in oxidoreductase activity and immune-related pathways, such as inflammatory response, and hydrolase activity post infection, whose function in the immune system have been reported in previous studies [34–38]. Oxidoreductases are enzymes that catalyze many redox reactions in normal cells [39]. Gostner et al. (2013) [34] indicated that redox reactions could initiate cytocidal reaction within the pathogen defense and trigger immune response, which are further involved in the process of cellular restorative. Xanthine oxidoreductase (XOR) has the capacity to produce both ROS and NO [35]. It is reported that the *A. hydrophila* infection affect ROS and NO reactive free radicals and induce an inflammatory response in zebrafish [36]. The iNOS is related to the immune response, which can induce NO to participate in immune regulation and anti-tumor mechanism [37]. Furthermore, the inducible isoform of nitric oxide synthase (iNOS) can be induced by cytokines such as interleukin, tumor necrosis factor, and interferon in the process of many diseases [37]. Triggering lyososomal rupture in dendritic cells might be to elevate intracellular ROS production for promoting antigen cross presentation [38].

In this study, KEGG pathway analysis showed that the target genes of the DE lncRNAs at different time points were mostly significantly enriched in the innate immune response pathways such as “adipocytokine signaling pathway”, “RIG-I-like receptor signaling pathway”, “herpes simplex infection”, and “Toll-like receptor signaling pathway”, which further confirmed that lncRNAs might participate in the regulation of fish immunity. Adipocytokine signaling pathway was one of the frequent pathways present in different time points comparisons in our study. It has been reported that the increase of genes expression related to various lipid and energy metabolism could reduce the inflammation in the body [40]. The peroxisome proliferator-activated receptors (PPARs) can modulate the expression of genes involved in lipid metabolism, maintenance of metabolic homeostasis, and inflammation [41]. After Toll-like receptors (TLR) or cytokine receptors in innate immune cells activate the intracellular signaling pathways to participate in immune responses, the expression of lncRNA in specific cell-types is induced [3]. This strongly indicates that these pathways play essential roles in the *M. amblycephala* response to *A. hydrophila* infection.

Obviously, we need more in-depth molecular mechanisms research to elucidate the specific function of lncRNAs to their target genes. The target genes of lncRNA in the module could indicate their biological processes and functions. Thus, we integrated lncRNAs and their target genes using WGCNA and BMKCloud (www.biocloud.net), and several highly correlated lncRNAs and mRNAs in six time-specific modules were identified. This genetic network may aid in functional analysis of lncRNAs, as most of them have not yet been identified as functional. These modules of co-expressed genes revealed the complex defense network, indicating diverse regulatory mechanisms of *M. amblycephala* post bacterial infection. For example, hepcidin was highly connected with 4 hpi (COR = 0.98) in the grey 60 module in the present study. We found that DEGs were significantly enriched in “iron ion binding” at 4 and 12 hpi. Previous studies have shown that there is a close connection between iron and innate immunity [42], and iron metabolism-related genes such as ferritin and transferrin have been verified to be related to immunity [43, 44]. Hepcidin is the main regulator of iron homeostasis and also the mediator of innate immunity [45]. The co-expressed lncRNAs related to hepcidin and other immune related genes in the iron metabolism pathway were analyzed by using BMKCloud and the co-expressed lncRNAs of those genes were selected for expression verification by RT-qPCR. The hepcidin and other immune related genes were up-regulated post bacterial infection, indicating that these genes played antibacterial immunity role after bacterial infection.

## Conclusions

This study explored the response of lncRNAs in the *M. amblycephala* liver to *A. hydrophila* infection. A total of 14,849 lncRNAs and 2196 DE lncRNAs were identified. GO and KEGG pathway analyses showed that the target genes of the DE lncRNAs were enriched in several pathways related to immune such as apoptosis, inflammation, and immune response. In addition, 28 time-specific modules were found by using WGCNA. Although the complexity of the natural environment cannot be fully explained, this research will enrich the lncRNAs database and contribute to a better understanding of the roles of lncRNAs in the immune response of teleost. Besides, since the lncRNAs can be a biomarker for human disease or a target for medical detection, we hope that our research on lncRNA could contribute to the research and detection of fish diseases. Further experimental studies are still needed on how lncRNAs work in the liver of *M. amblycephala*, and how it regulates the co-expressed genes involved.

## Materials and methods

### Samples collection

All the experimental procedures involved fish were approved by the Institutional Animal Care and Use Committee of Huazhong Agricultural University (Wuhan, China). The challenge experiment was conducted according to a previous study [44] with modification. In brief, unvaccinated healthy juvenile *M. amblycephala* (50 ± 10 g) were obtained from a fishery farm from Honghu city, Hubei province. The fish were kept in a recirculating freshwater system (temperature: 25–26 °C) and fed with a commercial pellet fish feed for 2 weeks before experimental manipulation. The experimental fish were injected intraperitoneally with 0.1 mL *A. hydrophila* (6 × 10^6^ CFU/mL). The liver tissues from 9 fish (3 pools with 3 fish per pool) at 0, 4, 12, 24, and 72 h post infection (hpi) were collected, respectively. Three liver samples were pooled as one biological duplicate and a total of 15 samples (3 biological duplicates at 5 time points) were used for later lncRNA-sequencing. The experimental fish were anesthetized with MS 222 at 100 mg/L before dissection. All samples were flash-frozen in liquid nitrogen and stored at − 80 °C for further use.

### LncRNAs library construction

After total RNA was extracted using TRIzol reagent (Invitrogen, CA, USA) according to the manufacturer’s instructions, its purity, concentration, and integrity were checked to ensure the use of qualified samples for transcriptome sequencing. rRNA was removed by using the Ribo-Zero rRNA Removal Kit (Epicentre, Madison, WI, USA). Sequencing libraries were constructed using the NEBNext^R^ Ultra™ Directional RNA Library Prep Kit for Illumina^R^ (NEB, USA) following the manufacturer’s recommendations. Insert fragments of 150–200 bp in length were purified with AMPure XP Beads (Beckman Coulter, Beverly, USA). Finally, the U chain was degraded, and the cDNA library was obtained by PCR enrichment. At last, PCR products were purified (AMPure XP system) and library quality was assessed using the Agilent Bioanalyzer 2100.

### Clustering, sequencing, and transcriptome assembly

The clustering of the index-coded samples was performed on acBot Cluster Generation System using TruSeq PE Cluster Kitv3-cBot-HS (Illumina, San Diego, CA, USA). After cluster generation, the library preparations were sequenced on Illumina NovaSeq 6000 (Illumina, San Diego, CA, USA). After sequencing, the Q20, Q30, GC-content and sequence duplication level of the clean data (clean reads) were calculated.

Our lncRNA detection pipeline started with aligning the timecourse RNA-seq paired-end reads from each time point to the *M. amblycephala* genome [46] using Hisat2 [47]. The transcriptome was assembled using the StringTie [48] based on the reads aligned to the reference genome and was used to calculate FPKM (Fragments per kilobase of transcript per million mapped reads) of both lncRNAs and coding genes in each sample. The assembled transcripts were annotated using the GffCompare program [49]. The filter criteria of lncRNAs transcripts were included in the “i” (transcripts entirely within intron), “x” (exonic overlap with reference on the opposite strand), “u” (intergenic transcripts), “o” (other same strand overlap with reference exons), and “e” (single exon transfrag partially covering an intron, possible pre-mRNA fragment) classes [49]. According to the above standard, transcripts longer than 200 nucleotide (nt) and that have two or more exons [50] were screened, and the FPKM was set ≥0.1. LncRNAs were screened using CPC2 [51]/ CNCI [52]/ Pfam [53]/ CPAT [54] that can distinguish the protein-coding genes from the non-coding genes.

### DEGs, DE lncRNAs analyses and functional annotation

Differential expression analysis of the library was performed using DEseq2 [55]. LncRNAs or protein-coding genes with a false discovery rate (FDR) < 0.05 and |log2 (Fold Change) | ≥ 1 were assigned as differentially expressed. For each lncRNA locus, the 100 kb upstream and downstream protein-coding genes (without overlap) were identified as cis-acting target genes. The trans-acting target genes of lncRNA were predicted by a correlation analysis method between the expression of lncRNA and mRNA. To analyze the main function of the mRNAs and lncRNAs, the genes were annotated through GO [56] and KEGG pathway analyses [57]. GO terms analysis was performed using the OmicShare tools, an online platform (http://www.omicshare.com/tools) for data analysis. The KEGG pathways analysis was performed using BMKCloud (www.biocloud.net). GO terms and KEGG pathways with *P*-values < 0.05 and the corrected *Q*-values < 0.05 were considered significantly enriched.

### Co-expression network analysis

Data were processed using the WGCNA package [58]. To ensure that the results of network construction are reliable, the abnormal values were removed. The soft threshold for network construction was selected to make sure that the network is closer to the real biological network state. The scale-free network was constructed using the blockwise modules function to group genes with similar patterns of expression.

The modules were defined by cutting the clustering tree into branches using a dynamic tree cutting algorithm and assigned to different colors for visualization [59]. To screen specific modules related to different time points post infection, the correlation coefficient *r* and corresponding *P*-value between the feature vector of each module and different time points were calculated. Time-specific modules were identified based on the correlation between gene significance (GS) and module membership (MM). Modules with significantly correlated GS and MM (*P* < 0.01) were defined as time-specific.

To further examine how lncRNAs cooperate with target genes to regulate fish immunity, co-expression analysis of the DE lncRNAs and the corresponding DE target genes was performed based on each time-specific module. Several differentially expressed immune-related mRNAs and predicted lncRNA-gene co-regulation pairs (COR > 0.8 and *P* < 0.01) were selected. Then Cytoscape 3.6 [60] was used to predict potential lncRNA targets with DEGs to build the lncRNA-gene co-expression network.

### Real-time fluorescent quantitative PCR (RT-qPCR) validation of lncRNA and gene expression

To verify the results of high-throughput RNA-seq, RT-qPCR was conducted. For the RT-qPCR analysis, 1 μg of total RNA was reverse transcribed using the RT reagent Kits with gDNA Eraser (Takara, Dalian, China) according to the manufacturer’s protocol. Six DE lncRNAs and four DEGs were chosen for RT-qPCR validation. Primer Premier 5.0 software was adopted to design the gene-specific primers (Additional file 7: Table S7).

The reaction of RT-qPCR was performed with CFX Connect Real-Time PCR Detection System (BIO-RAD, USA) according to standard methods using LightCycler@ 480 SYBR Green I Master (Roche, USA). *18 s rRNA* was used as an internal control [44]. All experiments were performed in triplicate. The amount of target molecules relative to the control was calculated by using the 2^-ΔΔCt^ method [61]. The results of RT-qPCR data were presented as mean ± standard error of the mean (SEM) and were calculated by SPSS 22.0 (SPSS Inc., Chicago, IL, USA). Differences between the control and experimental treatments were analyzed by one-way analysis of variance (ANOVA) through Dunnett’s multiple comparison. The level of statistical significance was set at *P* < 0.05, and highly significance at *P* < 0.01.

## Supplementary Information

**Additional file 1: Supplementary Table 1**. Statistical table of sample sequencing data evaluation.

**Additional file 2: Supplementary Table 2**. Correlation of repeated samples.

**Additional file 3: Supplementary Table 3**.The target genes of lncRNAs.

**Additional file 4: Supplementary Table 4**. GO analysis of the differentially expressed genes.

**Additional file 5: Supplementary Table 5**. GO analysis of the target genes of differentially expressed lncRNAs.

**Additional file 6: Supplementary Table 6**. KEGG analysis of the target genes of differentially expressed lncRNAs.

**Additional file 7: Table S7** Primers used for RT-qPCR.

## Data Availability

The sequencing data in this study were deposited in the Seq Read Archive (SRA) database (accession number: PRJNA716731) at https://www.ncbi.nlm.nih.gov/bioproject/PRJNA716731.

## Abbreviations

LncRNAs: Long non-coding RNAs
bp: Base-pairs
GO: Gene Ontology
KEGG: Kyoto Encyclopedia of Genes and Genomes
WGCNA: Weighted correlation network analysis
RT-qPCR: Real-time fluorescent quantitative PCR
ceRNA: Competing endogenous RNA
miRNAs: MicroRNAs
MREs: MicroRNA response elements
AID: Autoimmune and immune-related disorders
IL-1β: Interleukin-1β
XOR: Xanthine oxidoreductase
iNOS: Inducible isoform of nitric oxide synthase
PPARs: Peroxisome proliferator-activated receptors
TLR: Toll-like receptors
hpi: Hour post infection
lincRNA: Intergenic lncRNA
FPKM: Fragments per kilobase of transcript per million mapped reads
nt: Nucleotide
DEGs: Differential expression genes
DE lncRNAs: Differentially expressed lncRNAs
FDR: False discovery rate
GS: Gene significance
MM: Module membership
SEM: Mean ± standard error
