# Supplementary material for: Integrated analysis of lncRNA and mRNA in liver of Megalobrama amblycephala post Aeromonas hydrophila infection
Source: BMC Genomics. 2021 Sep 11;22:653. doi: 10.1186/s12864-021-07969-5 (PMC8435129; doi:10.1186/s12864-021-07969-5)
Supplement: Supplementary file 7 — Additional file 7: Table S7 Primers used for RT-qPCR. [file 12864_2021_7969_MOESM7_ESM.docx]

**Additional file 7: Table S7 Primers used for RT-qPCR**

**Table S7 Primers used for RT-qPCR**

| Name of primer | Primer Sequence（5’-3’） |
| --- | --- |
| MSTRG.5748.1-F | 5' CAGCTCTGCAATTCAGCACTCAA 3' |
| MSTRG.5748.1-R | 5' AAGAAAAGCAAGCAAAACAACCA 3' |
| MSTRG.19331.1-F | 5' ATGAAGTGGAGTTTCCCAGTTG 3' |
| MSTRG.19331.1-R | 5' TTATTAGACAGTCGCTGAGGGG 3' |
| MSTRG.55000.1-F | 5' TGACGTGATATTTGGGGTAGTTAG 3' |
| MSTRG.55000.1-R | 5' AGCCTGAAAACACGAACACATT 3' |
| MSTRG.81802.1-F | 5' AGAAATCCGATAGCGTGGTT 3' |
| MSTRG.81802.1-R | 5' ATGTCTATACACACTTATCCAGCCT 3' |
| MSTRG.78538.13-F | 5' GTGCCTCATCCCAGACCTCTTATT 3' |
| MSTRG.78538.13-R | 5' TTGGCTGAAACTATACTACACCCC 3 |
| MSTRG.64203.2-F | 5' CAGGCACTAATCTGAACCAGG 3' |
| MSTRG.64203.2-R | 5' CATTCCCAGTAATCCGCAC 3' |
| hepcidin-F | 5'CAGACCGCAGCCGTTCCCTT 3' |
| hepcidin-R | 5'AGCAGTATCCACAGCCTTTG 3' |
| Transferrin-F | 5'TTGTGGCGGTTGTGCGTAA 3' |
| Transferrin-R | 5'CCTGGAAGCCCCATCATAGC 3' |
| Ferritin-M-F | 5'TTCAAGGAGAACAGCGAGGAGG 3' |
| Ferritin-M-R | 5'CCAGGAAGTCACACAGATGAGGGT 3' |
| FPN-F | 5' GAGGAAGCCCCCCAAGTAGA 3' |
| FPN-R | 5' ATGATCACGAGAAGGCCGAA 3' |
| q18S rRNA-F | 5' CGGAGGTTCGAAGACGATCA 3' |
| q18S rRNA-R | 5' GGGTCGGCATCGTTTACG 3' |
